# Systemic deficiency of GM1 ganglioside in Parkinson’s disease tissues and its relation to the disease etiology

**DOI:** 10.1007/s10719-021-10025-9

**Published:** 2022-01-01

**Authors:** Robert Ledeen, Suman Chowdhury, Zi-Hua Lu, Monami Chakraborty, Gusheng Wu

**Affiliations:** Department of Pharmacology, Physiology & Neuroscience, Rutgers The State University of New Jersey, Newark, NJ 07103 USA

**Keywords:** Parkinson’s disease, GM1 ganglioside, Multi-system disorder, Subnormal GM1, Aging induced subnormal GM1

## Abstract

Following our initial reports on subnormal levels of GM1 in the substantia nigra and occipital cortex of Parkinson’s disease (PD) patients, we have examined additional tissues from such patients and found these are also deficient in the ganglioside. These include innervated tissues intimately involved in PD pathology such as colon, heart and others, somewhat less intimately involved, such as skin and fibroblasts. Finally, we have analyzed GM1 in peripheral blood mononuclear cells, a type of tissue apparently with no direct innervation, and found those too to be deficient in GM1. Those patients were all afflicted with the sporadic form of PD (sPD), and we therefore conclude that systemic deficiency of GM1 is a characteristic of this major type of PD. Age is one factor in GM1 decline but is not sufficient; additional GM1 suppressive factors are involved in producing sPD. We discuss these and why GM1 replacement offers promise as a disease-altering therapy.

**Supplementary information:**

The online version contains supplementary material available at 10.1007/s10719-021-10025-9.

## Introduction

Parkinson’s disease (PD) was earlier viewed primarily as a movement disorder involving the nigral dopaminergic (DA) neurons of the substantia nigra pars compacta (SNpc) but has gradually come to be recognized as a multi-system disease involving virtually all neurons of the central nervous system (CNS) and peripheral nervous system (PNS). We have been interested in the role of GM1 ganglioside, considering its subnormal expression found in the brain and have considered this in relation to the diverse pathophysiological manifestations of this complex disorder. We first employed immunohistochemistry to observe significantly deficient GM1 in the DA neurons of the SNpc of PD patients—as well as in adjacent cells, the latter giving us the first clue that this deficiency was not limited to DA neurons of the SNpc [1]. We subsequently employed high-performance thin layer chromatography (HPTLC) combined with cholera toxin B (CtxB) subunit to demonstrate a similar deficiency in the occipital cortex [2], another part of the CNS albeit less intimately involved in overt PD symptoms. The present study attempts to extend those findings by showing that non-CNS tissues also express reduced levels of GM1, indicating such subnormal expression of GM1 to be systemic and thus correlated with the numerous motor and non-motor pathologies of this disorder.

Pathophysiological disabling of neurons outside the CNS was well revealed in the ground-breaking studies of Braak and coworkers using Lewy pathologies as the chief neuropathological indicator [3–5] and by the work of others [6]. Such findings were effectively correlated with various non-motor symptoms of PD. The discovery that aSyn aggregates can propagate from cell-to-cell in a prion-like manner [7] led to the question of disease origin and the proposal that PD comprises two subtypes, a brain-first form with aSyn aggregates migrating from the brain to peripheral sites, and a body-first type with initial pathology in the enteric or peripheral autonomic nervous system with migration in the opposite direction [8]. We favor an alternative mechanism to explain the diverse manifestations of PD pathology based on GM1 deficiency throughout the brain and body (see Discussion). The present study provides supporting data for this hypothesis.

## Materials and methods

Tissues from PD patients and age-matched controls were obtained from the Brain and Body Donation Program of the Banner Sun Health Research Institute (Sun City, AZ; Dr. Thomas Beach). The tissues were cut into small pieces and homogenized in chloroform (C): methanol (M) (1:1; by vol) to extract total lipids. Proteins and other non-lipids were pelleted by centrifugation and the C-M-lipid supernatant was removed. This was evaporated to dryness, reconstituted in a smaller volume of C-M, and applied to HPTLC plates, which were developed in C/M/0.25 M KCl) as described [2, 9]. GM1 was detected with CtxB-HRP and the other ganglio-series gangliosides in the same way following conversion of these to GM1 via application of neuraminidase (N’ase) to the plate. It was not necessary to separate gangliosides from the other extracted lipids because the latter migrated well ahead on the HPTLC and did not interfere with ganglioside migration and detection.

Fibroblasts of PD patients and control subjects were obtained from Cornell Institute for Medical Research (Camden, NJ). They were cultured in DMEM with 10% fetal bovine serum (FBS). For cytochemical staining of GM1, cells were seeded onto glass coverslips placed in multiwell plates in the same medium. Cells were allowed to grow for four to five days and then fixed in 2% paraformaldehyde in PBS. GM1 expression was detected by incubation with CtxB-FITC (1 µg CtxB/ml) at room temp for 30 min. FITC fluorescence intensity was measured from 0 to 4095 [2]. Statistical significance was determined by Student's t-test.

Peripheral blood mononuclear cells (PBMCs) were a kind gift from Dr. Roy Alcalay, Dept. of Neurology, Columbia University, New York, NY). These included, in addition to normal controls., sporadic PD (sPD) and the glucocerebrosidase variant (PD-GBA). These were treated with small volumes of C-M and processed as above. The amounts of lipid extracts applied to the HPTLC plates were normalized according to the protein content—remaining after C-M extraction [9, 10]. Following HPTLC development and CtxB-HRP application, GM1 and the other gangliosides were revealed by ECL reagent and quantified by densitometry.

## Results

**Fig. 1:**
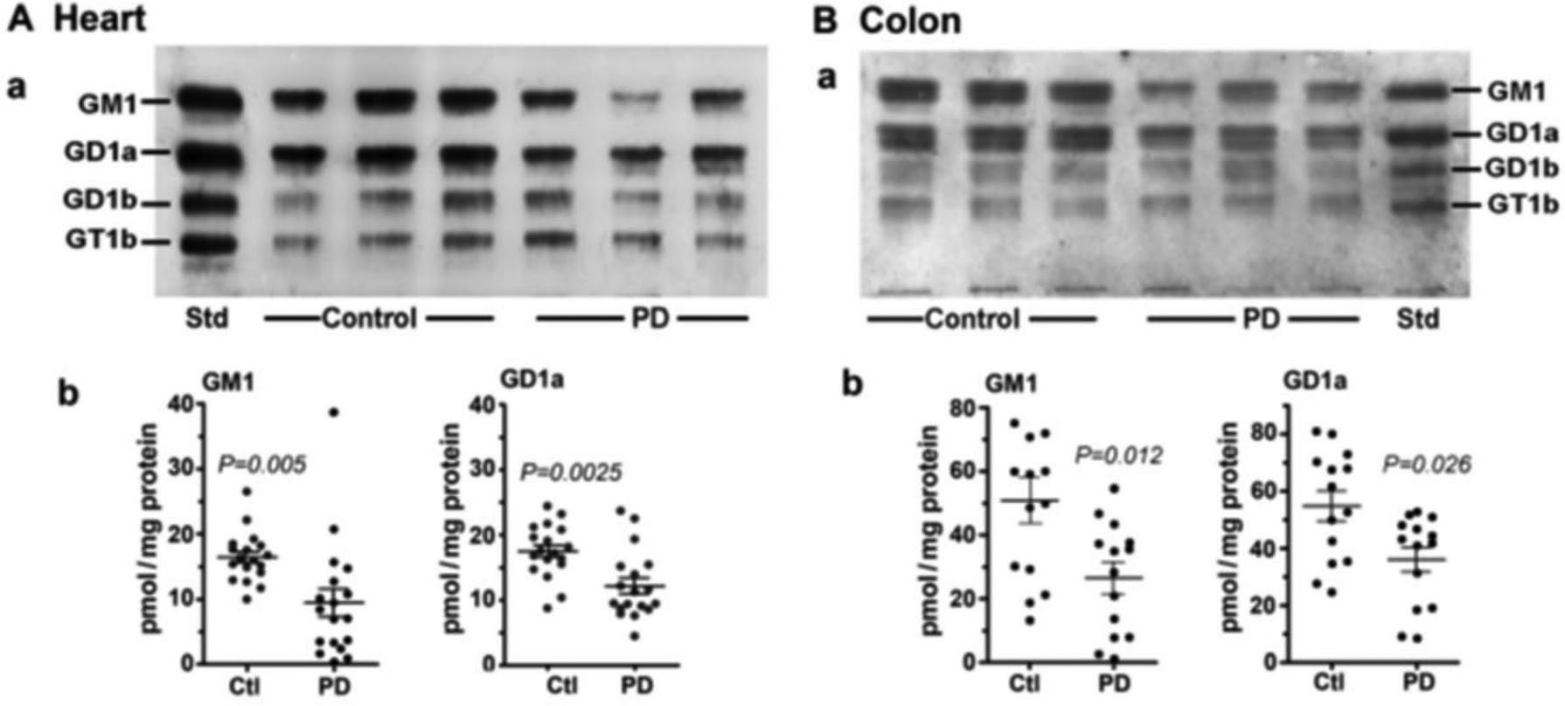
Gangliosides in heart and colon from PD patients and age-matched controls. **A**: Heart (n = 18 in each group), **B**: Colon (n = 14 in each group); In **A-B**, subpanel **a** is HPTLC, and subpanel **b** is densitometry quantification showing the statistical difference between PD and controls, determined by Mann–Whitney rank sum U test

We have first focused on tissues well documented to show pathological involvement in PD, starting with the gastrointestinal system- e.g., colon. This was demonstrated by Braak and coworkers, employing aSyn immunoreactive inclusions (see above). Neurocardiology is another system showing early onset of pathological damage in PD. As part of the autonomic nervous system, heart tissue suffers a loss of sympathetic innervation, [11]. As before, aSyn aggregates constituted the principal diagnostic tool [12]. Both colon and heart showed a significant deficiency of GM1 compared to normal controls (Fig. 1). Typical HPTLC results are shown in addition to GM1 quantification. Also shown is a similar result for GD1a, the other member of the ganglio-series gangliosides that showed subnormal expression in PD. GD1a has assumed importance as a reservoir for GM1 since it serves as a metabolic precursor to GM1 via the action of the NEU3 form of N’ase which is situated close to GD1a in the membrane [13].

**Fig. 2:**
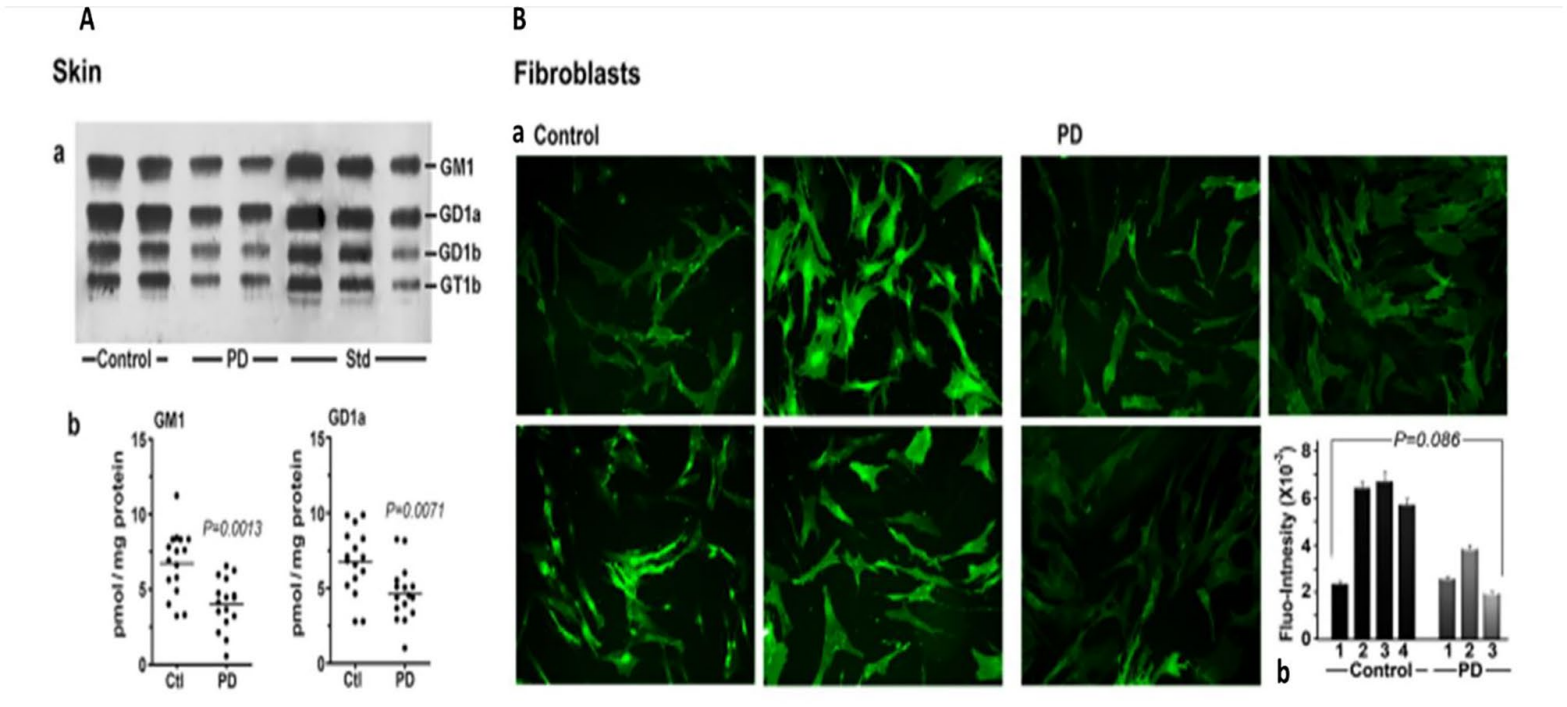
Gangliosides in skin and fibroblasts from PD patients and age-matched controls. **A**: Skin (n = 16 in each group), Subpanel **a** is HPTLC, and subpanel **b** is densitometry quantification showing significant difference between PD and controls, calculated by Mann–Whitney rank sum U test. **B**: **a** GM1-fluorescent images of cultured fibroblast cells from four non-PD controls and three age-matched PD patients were immunostained for GM1; **b** fluorescence intensity of GM1 staining in fibroblast cells, showing a marginal difference in GM1 by Student's t-test

Our attention next turned to the skin; a tissue sometimes overlooked as among those involved in the non-motor symptoms. It was early recognized as representing a pre-motor (prodromal) feature of PD along with other pathologies of the autonomic nervous system [14]. Seborrheic dermatitis is a principal manifestation, appearing in the head and neck areas as well as the upper trunk and sternum. HPTLC analysis of skin lipids indicated a significant deficiency of GM1 (Fig. 2a, b), while fluorescent immunohistochemical analysis of cultured fibroblasts also showed GM1 deficiency (Fig. 2c); however, this did not quite reach significance, likely due to the limited number of samples available.

**Fig. 3:**
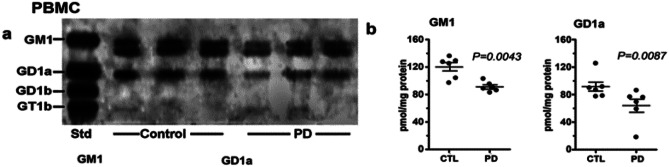
Gangliosides in PBMCs from PD patients and age-matched controls. These were analyzed with HPTLC (n = 6 in each group). Subpanel **a** is HPTLC, and subpanel **b** is densitometry quantification showing the statistical difference between PD and controls, calculated by Mann–Whitney rank sum U test

More recently we have focused on PBMCs which show similar GM1 depletion as found in neurons (Fig. 3). Despite their lack of direct neuronal involvement, these white blood cells show a reduction of intracellular DA, tyrosine hydroxylase, and DA transporter [15, 16]. Here too we found GD1a as well as GM1 to be depleted. In a more detailed study of PBMCs, we recently found such GM1 deficiency to be more pronounced in PD patients with the glucocerebrosidase (GBA) malfunction than in those with the more common form of sPD [17], similar to the result in Supplementary File Fig. S1. We have proposed GM1 deficiency as a potential tool for early diagnosis of the two forms of PD (see Discussion).

## Discussion

These results on GM1 deficiency in non-CNS PD tissues, together with our similar findings in the CNS [1, 2], support the hypothesis that GM1 manifests systemic deficiency in virtually all tissues of the PD patient’s body. That this included white blood cells (PBMCs) was unexpected since such cells do not appear to participate directly in neural functioning. However, as mentioned, these cells are known to contain DA, tyrosine hydroxylase, and the DA transporter, which also declined in PD [18]. The latter study showed significant GM1 reductions in substantia nigra, CSF, and serum, which also supported the idea of systemic deficiency of GM1. This occurred despite increases in other glycosphingolipids stemming from reduced activities of various lysosomal hydrolases—including some that might be expected to elevate GM1 (e.g., glucocerebrosidase).

This proposed body-wide deficiency correlates well with the broad array of CNS and peripheral symptoms that characterize PD revealed by Braak *et al.* (see above) and others more recently [6, 19, 20]. We have accordingly proposed subnormal GM1 as a major risk factor in sPD. This pertains to the 90% (or more) of PD cases in which the underlying etiology is unknown (sPD), in contrast to the approximately 10% of PD cases based on hereditary forms of PD emanating from specific genetic mutations [21].

## Cause of GM1 deficiency: age plus additional suppression

The question then arises as to the cause of deficient GM1 in PD. An important part of the answer is undoubtedly the aging process itself, in keeping with a key discovery by Svennerholm and coworkers that GM1 and GD1a decrease progressively with age [22]. This decrease was particularly notable for GD1a, the above-mentioned metabolic precursor of GM1 [23]. Svennerhom and coworkers also noted significant differences in GM1/GD1a between individuals of the same age. However, it is important to note that age-related decreases alone would not explain the total difference, considering that age-matched controls used for comparison to PD represent individuals with Svennerholm-like decreases in GM1 and GD1a without the development of PD. In like manner, Huebecker *et al.* showed age-related decreases in GM1 and GD1a in the substantia nigra of normal controls at approximately 60—90 years of age without accompanying disease [18].

In other words, there must be an additional factor(s) that depresses GM1 and GD1a even further to correspond to the highly depressed pathological levels in the disease. One such additional factor might well be defective lysosomal hydrolase activity of one type or another, as seen in our results with PBMC. Glucocerebrosidase in the latter example is the most prevalent of these but it may be relevant that excessive burden of potentially damaging variants of 50 or more lysosomal storage disorder genes has been reported in PD cases [24]. Lysosomal dysfunction has been linked to PD in a number of ways, but of critical importance to PD is aSyn degradation which is mostly lysosomal dependent [25]; lysosomal dysfunction in this process can lead to aSyn accumulation and aggregation. It is noteworthy that GM1 has been described as promoting autophagy-dependent removal of aSyn in a PD mouse model [26].

An additional factor worthy of consideration concerning the subnormal level of GM1 is the microbiome, recently studied in relation to a PD model [27]. A study of actual PD patients revealed significantly reduced fecal short-chain fatty acids, a metabolic product of certain gut bacteria that were also deficient [28]. Short-chain fatty acids are known to inhibit histone deacetylase [29, 30], a process shown to promote epigenetic activation of GM1 synthesis [31, 32].

Yet additional factors potentially functioning as GM1/GD1a suppressors are environmental neurotoxins [33]. Preliminary studies of Morrison *et al.* showed that low-level pesticide exposure can decrease GM1 in otherwise healthy DA neurons [34].

To understand abnormal GM1 Niimi *et al.*, demonstrated glucosylceramide level is significantly downregulated in PD, and suggested GM1 reduction in CNS results from increase in its degradation rather than decrease in its production; no reduction of precursor for GM1 was observed [35]. In another study Niimi *et al.*, observed higher β-Galactosidase (β-Gal) activity in blood serum of PD patients than in normal control group. In normal group, a negative correlation was seen between age and β-Gal activity whereas a positive correlation was seen in PD patients. This upregulation of β-Gal activity, which is responsible for breakdown of GM1 in lysosomes was proposed as an explanation of why GM1 decreases in the PD patients [36]. This possibility is certainly worth further exploration.

## Simultaneous decrease of GM1 in brain and body

It is important to recognize that the progressive decline of GM1 is occurring not just in the brain but throughout the body—to varying degrees among the diverse tissues and between individuals. Hence there is no need to postulate prion-like migration of aggregated aSyn or to contemplate brain first vs body first progression. Such prion-like transfer very likely does occur but most probably on a limited scale, for it is difficult to see how this would account for PD manifestation in such diverse locations as gastrointestinal, cardiological, and dermatological localized neurons, among others.

## Critical role of GM1 and alpha-synuclein

The primary mechanism underlying this essentiality of GM1 is its association with the several proteins that require such binding to maintain their appropriate conformation and related function [37, 38]. A prime example, of special relevance to PD, is aSyn, whose aggregation stems from conformational loss and resultant misfolding. This is generally viewed as a major contributor to neuronal loss and PD pathology. Alpha-synuclein binds GM1 with high affinity and specificity, thereby retaining it in alpha-helical, non-aggregating conformation [39, 40]. This was well-demonstrated *in vivo* by the dispersing of aSyn aggregates via the application of GM1 to mice expressing such aggregates [1, 41].

The functional role of aSyn is not entirely clear, but it was described as an almost entirely soluble protein in brain extracts [42]. If aSyn is largely soluble and intraneuronal, that raises the question, how does it maintain its monomeric, helical conformation to avoid aggregation? We see this as an important function of intraneuronal GM1, in particular the small pool of cytosolic, soluble GM1 which was shown to bind soluble proteins (not yet identified) [43, 44]. We have proposed this pool of soluble GM1 to exist in stoichiometric balance with soluble aSyn, which binds to GM1 with high affinity and specificity [39, 40]. Preliminary evidence for this mechanism has been revealed in the aggregation of aSyn following depletion of GM1 in cultured NG108-15 neuroblastoma cells [see Supplementary File Fig. 2]. Importantly, such aSyn-GM1 balance would be susceptible to disruption due to even partial deficiency of GM1, leading to the gradual accumulation of unassociated aSyn with resultant aggregation and PD pathologies.

## Critical role of GM1 and neurotrophic factors

Another GM1-associated function, conceivably as relevant to PD as aSyn, is the GM1 activation of glial cell line-derived neurotrophic factor (GDNF). Our study of both the mouse model and PD tissues revealed GM1 as an obligatory component of the duo-protein receptor complex of this important neurotrophic factor (NTF) [2]. GDNF was shown necessary for the long-term viability of adult catecholaminergic neurons, including those of the SNpc [45]. Notably, receptors for NGF and BDNF, two other major NTFs, are also tightly associated with GM1 which is required for their neuroprotective function [46, 47]. This points to the broad array of neurons dependent on these NTFs with their associated GM1 that are destined to gradually dysfunction and die, albeit at different rates, in the absence of adequate GM1.

## Additional functions of GM1

Several other essential functions have been attributed to GM1 which, along with other members of the ganglio-series, occurs with special abundance in neurons of the CNS and PNS and is essential for their long-term survival [37, 38]. One example is calcium (Ca) transport mediated at both the nuclear and plasma membranes by GM1 associated with a sodium (Na)-Ca exchanger [48] or an integrin, protein which opens a TRPC5 channel [49]. Some G-protein coupled receptors are regulated by GM1, one interesting example being the delta-opioid receptor [50]. There are thus multiple reasons why neuron function and survival depend on an adequate level of GM1 and GD1a, its reserve pool.

## Mouse model and human parallel

Our contention that subnormal GM1 is the cause (rather than the result) of systemic PD is strongly supported by the mouse PD model based on mono-allelic inactivation of the B4galnt1 (GM2 synthase) gene, resulting in partial deletion of GM1 throughout the brain and body [1, 2, 51]. Such mice, without the use of a neurotoxin, show the neuropathological characteristics of PD together with motor dysfunctions related to the CNS [1, 2] and non-motor symptoms in the periphery [51]. The success with this mouse model led to speculation that PD itself may have a similar genetic cause, although genome-wide association studies revealed no evidence that B4galnt1 or other GM1 synthesis genes are affected in PD [52]. This was true despite an *in-situ* hybridization study that showed a significant reduction of B3galt4 (GM1 synthase) and ST3gal2 (GD1a synthase) specifically in a neuromelanin-containing cells of the substantia nigra of PD patients [53]. However, B4galnt1 (GM2 synthase) was apparently not included in the study. We consider it possible the answer may lie in epigenetic effects, previously shown capable of influencing ganglioside expression [31, 32]. Naturally occurring disruption of the B4galnt1 gene has been reported [54, 55]. Although one such study described this as a relatively mild disorder, the other two described it as complex hereditary spastic paraplegia. This condition, along with mutations in St3gal5 (GM3 synthase) occurs sporadically but is especially prevalent in the Amish community, which has been reported to have one of the world’s highest incidences of PD [56, 57]. The latter may be related to the likelihood of GM1 deficiency in first degree relatives of the afflicted children, and such relatives would be heterozygous carriers of GM1 deficiency.

## GM1 as a therapeutic solution to Parkinson's disease

The above findings on subnormal GM1 as a major risk factor for sPD point to GM1 replacement as potential disease altering therapy. This was implied in the successful results with various animal PD models showing marked improvement with administered GM1 as summarized in Ledeen *et al*., 2018 [58]. Additional strong evidence comes from the clinical trials conducted by Jay Schneider and coworkers. One of these was a five-year open-label study beginning with an initial "loading dose" of 1000 mg GM1 followed by two daily doses of 100 mg of GM1 administered subcutaneously [59]. This resulted in improvement on UPDRS motor scores, these showing less disability after five years than at baseline. This was followed by a randomized, controlled, delayed-start phase II trial resulting in reduced motor impairment and slowing of symptoms over a two-year period [60]. UPDRS scores were improved at the trial end of 120 weeks, all this suggesting to the investigators that "GM1 may have symptomatic and potentially disease-modifying effects".

That more striking benefits were not in evidence could like to be attributed to the limited ability of GM1 to enter the brain and gain intracellular access to neurons of the CNS and PNS. Studies are in progress to resolve this limitation. The above clinical trials were conducted with GM1 from the animal brain (e.g., bovine) and were terminated by the FDA due to fear of possible contamination by prion-like proteins. The use of E. coli-derived GM1 proved equally as effective as bovine-derived GM1 in a mouse PD model [51] but this too was terminated due to financial problems by the involved pharmaceutical company. However, the promise offered by the above results argues strongly in favor of continuing this line of investigation as a promising solution to the devastating and growing pandemic of PD among the diverse populations of the world.

## Supplementary information

Below is the link to the electronic supplementary material.

Supplementary file1 (DOCX 1.32 MB)

## Data Availability

Data will be made available on reasonable request.

## Abbreviations

PD: Parkinson’s disease
DA: Dopaminergic
SNpc: Substantia nigra pars compacta
CNS: Central nervous system
PNS: Peripheral nervous system
HPTLS: High-performance thin layer chromatography
CtxB: Cholera toxin B
C: Chloroform
M: Methanol
N’ase: Neuraminidase
FBS: Fetal bovine serum
PBMCs: Peripheral blood mononuclear cells
sPD: Sporadic PD
PD-GBA: Glucocerebrosidase variant PD
GBA: Glucocerebrosidase
GDNF: Glial cell line-derived neurotrophic factor
NTF: Neurotrophic factor
Ca: Calcium
Na: Sodium
