# Supplementary material for: Systemic deficiency of GM1 ganglioside in Parkinson’s disease tissues and its relation to the disease etiology
Source: Glycoconj J. 2022 Jan 1;39(1):75–82. doi: 10.1007/s10719-021-10025-9 (PMC8979856; doi:10.1007/s10719-021-10025-9)
Supplement: Supplementary file 1 — Supplementary file1 (DOCX 1.32 MB) [file 10719_2021_10025_MOESM1_ESM.docx]

**Systemic deficiency of GM1 ganglioside in Parkinson’s disease tissues and its relation to the disease etiology**

**Robert Ledeen^1*^ · Suman Chowdhury^1^ · Zi‐Hua Lu^1^ · Monami Chakraborty^1^ · Gusheng Wu^1^**

*Correspondence:

Dr. Robert Ledeen- ledeenro@njms.rutgers.edu

Department of Pharmacology, Physiology, and Neuroscience, Rutgers, The State University of New Jersey, Newark-07103, New Jersey, USA

**
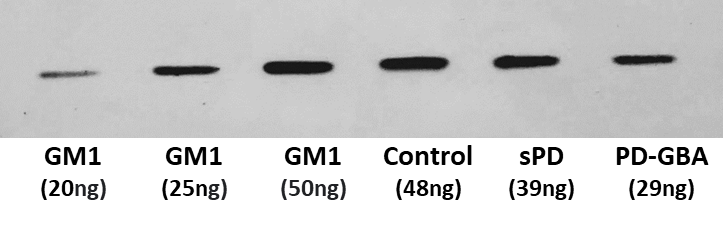
**

Fig. S1 Example of HPTLC detection of GM1 derived from PBMCs of healthy controls, sPD, PD-GBA patients and varying concentrations of GM1 standard (see Discussion).

**Protocol for the Immunofluorescence**

# Materials

# Cholera Toxin B subunit-(FITC) conjugate (Cat# C1655, Sigma), Rabbit monoclonal anti-alpha-synuclein aggregate (Cat# ab209538, Abcam) and Goat anti-Rabbit IgG (H+L) secondary antibody, Texas Red-X (Cat# T6391, Invitrogen) were purchased from Sigma, Abcam and Invitrogen, respectively. D-threo-1-phenyl-2-Palmitoylamino-3-morpholino-1-propanol (PPMP) was from Cayman chemical (USA) and 4',6-Diamidino-2-Phenylindole, Dihydrochloride (DAPI) was purchased from Invitrogen, USA. All the other chemicals used were from Sigma, USA.

# Method

# NG108-15 cells were cultured on poly-d-lysine coated coverslips in 12-well plates and treated with D-PPMP at 20 uM final concentration for 48 hrs. The cells were then fixed for 15 min at room temperature in 2% paraformaldehyde (in PBS at pH 7.4), followed by washing with 1X PBS for 5 min. The cells were then permeabilized and simultaneously blocked for 60 min in PBS + 0.1% Triton X-100 + 10% FBS solution. Primary antibodies were diluted in PBS + 0.1% Triton X-100 + 10% FBS solution (Cholera Toxin B subunit-(FITC) conjugate, 1.5 ug/mL; anti-alpha-synuclein aggregate, 0.2 ug/mL) and incubated together with permeabilized cells overnight at 4°C. Next day, antibody solution was removed, and cells were washed with 1X PBS twice for 5 min. For the binding of secondary antibodies conjugated with Texas red (Goat anti-Rabbit IgG (H+L) secondary antibody, Texas Red-X, 5 ug/mL) cells were incubated for 2 hrs at room temperature before being washed twice for 5 min with 1X PBS. Cells were further incubated with DAPI (0.5 ug/ml), for nuclear staining for 15 min at room temperature, followed by two washing with 1X PBS for 5 min. For mounting coverslips on slides, fluoroshield medium was used, and images were acquired on an Olympus BX51 microscope, using a 40X objective.

# Result

# To study the effect of the inhibitor of glycosphingolipids biosynthesis on GM1 expression on neuroblastoma cell line, NG108-15 cells were cultured for 48 hrs in DMEM media (with 10% FBS) with or without the PPMP inhibitor. PPMP is an endogenous glucosylceramide synthase inhibitor refraining biosynthesis of glycosphingolipids (GSL), which ultimately leads to depletion of GM1. Fluorescent staining showed significant decrease in GM1 along with an increase in aggregated aSyn expression. In comparison, untreated (control) NG108-15 cells showed high expression of GM1 and negligible aggregated aSyn. The increase in aggregated aSyn in PPMP treated NG108-15 cells is attributed to decrease in endogenous GM1 required to bind to aSyn for retaining it in monomeric, non-aggregated state.


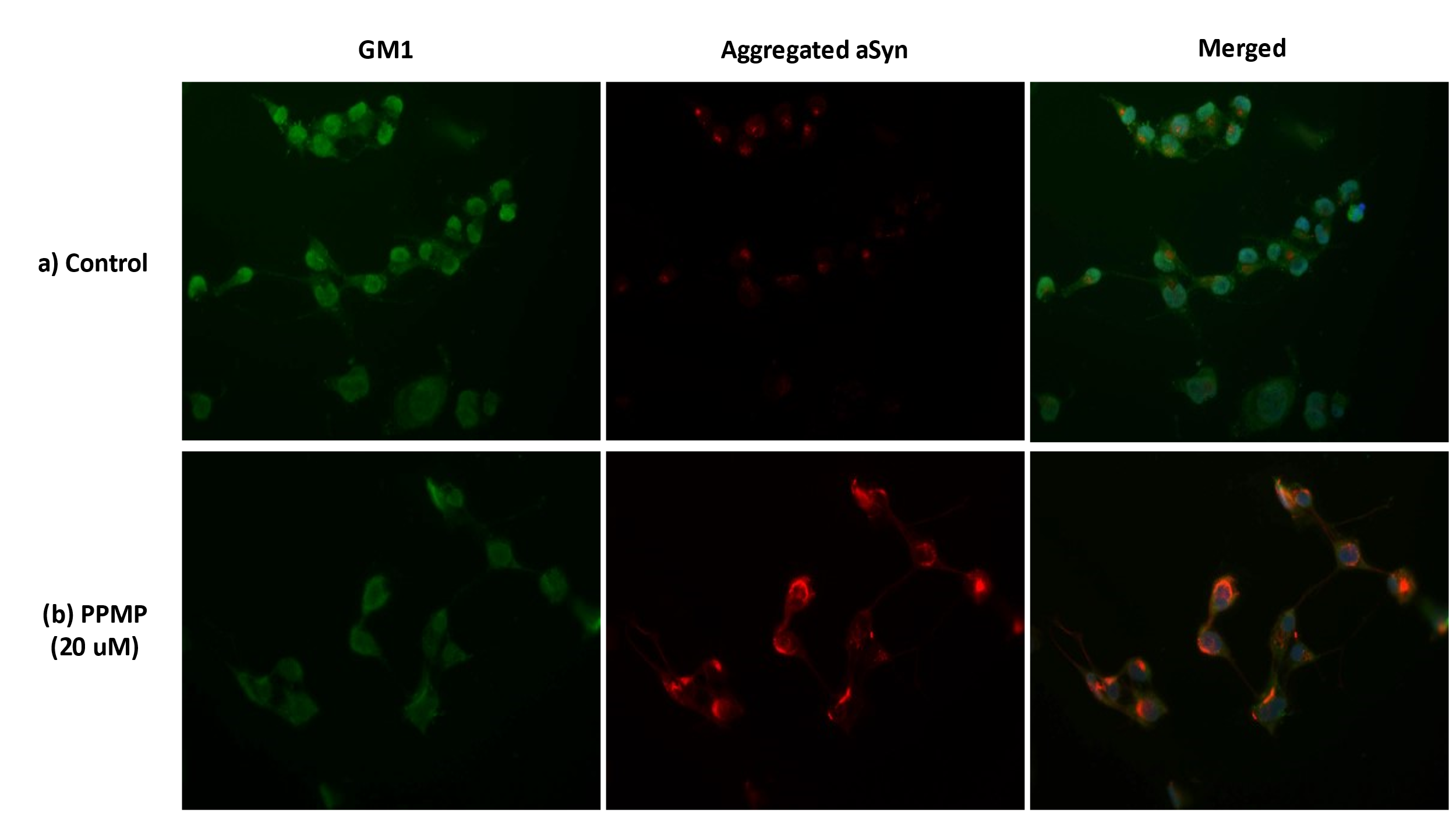


Fig. S2. Fluorescent image analysis of double-labeled NG108-15 cells treated as described above. Note increase in aggregated aSyn resulting from PPMP-induced reduction in GM1.

# Conclusion

Alpha-synuclein is largely found in the soluble state in neuronal cytosol, which requires the monomeric, alpha-helical form of the protein. It has been reported that this soluble GM1 is bound to soluble proteins, and we hypothesize that one such protein is aSyn. The above preliminary study targeted cytosolic GM1 in neuroblastoma NG108-15 cells by addition of anti-aSyn antibody, following fixation and very mild permeabilization. The above result indicates that cytosolic GM1 coexists with soluble aSyn in mandatory binding. However, with depletion of cytosolic GM1 and the absence of aSyn-GM1 association, aSyn converts predominantly to the aggregated form. Hence, aSyn is dependent on GM1 association to maintain its alpha-helical conformation and remain a soluble, monomeric protein.
